# Efficient *In Silico* Identification of a Common Insertion in the *MAK* Gene which Causes Retinitis Pigmentosa

**DOI:** 10.1371/journal.pone.0142614

**Published:** 2015-11-11

**Authors:** Kinga M. Bujakowska, Joseph White, Emily Place, Mark Consugar, Jason Comander

**Affiliations:** Ocular Genomics Institute, Massachusetts Eye and Ear Infirmary, Harvard Medical School, Boston, Massachusetts, United States of America; Hadassah-Hebrew University Medical Center, ISRAEL

## Abstract

**Background:**

Next generation sequencing (NGS) offers a rapid and comprehensive method of screening for mutations associated with retinitis pigmentosa and related disorders. However, certain sequence alterations such as large insertions or deletions may remain undetected using standard NGS pipelines. One such mutation is a recently-identified Alu insertion into the *Male Germ Cell-Associated Kinase* (*MAK*) gene, which is missed by standard NGS-based variant callers. Here, we developed an *in silico* method of searching NGS raw sequence reads to detect this mutation, without the need to recalculate sequence alignments or to screen every sample by PCR.

**Methods:**

The Linux program *grep* was used to search for a 23 bp “probe” sequence containing the known junction sequence of the insert. A corresponding search was performed with the wildtype sequence. The matching reads were counted and further compared to the known sequences of the full wildtype and mutant genomic loci. (See https://github.com/MEEIBioinformaticsCenter/grepsearch.)

**Results:**

In a test sample set consisting of eleven previously published homozygous mutants, detection of the *MAK*-Alu insertion was validated with 100% sensitivity and specificity. As a discovery cohort, raw NGS reads from 1,847 samples (including custom and whole exome selective capture) were searched in ~1 hour on a local computer cluster, yielding an additional five samples with *MAK*-Alu insertions and solving two previously unsolved pedigrees. Of these, one patient was homozygous for the insertion, one compound heterozygous with a missense change on the other allele (c. 46G>A; p.Gly16Arg), and three were heterozygous carriers.

**Conclusions:**

Using the *MAK*-Alu *grep* program proved to be a rapid and effective method of finding a known, disease-causing Alu insertion in a large cohort of patients with NGS data. This simple approach avoids wet-lab assays or computationally expensive algorithms, and could also be used for other known disease-causing insertions and deletions.

## Introduction

The genetics of retinitis pigmentosa (RP) is particularly challenging due to the large numbers of genes that can cause similar clinical phenotypes [1–3]. Even though it is usually a monogenic, Mendelian disorder, over 90 genes are associated with RP and related disorders [3]. For this reason, the use of NGS has allowed for more comprehensive analysis of these genes and is becoming more widespread for clinical testing [4,5]. However, recent experience shows that there are some regions of the genome that are difficult to analyze by NGS, due to GC-rich highly repetitive sequence or deep intronic mutations not captured by standard NGS techniques [4,6–8]. In addition, the overall diagnostic success rate for retinitis pigmentosa is about 50% [4,9–11] suggesting unknown disease genes or “missing inheritance” in the known disease-associated genes.

Despite much effort, detection of large deletions and insertions (indels) from next generation sequencing (NGS) data is still a challenging problem. Most methods fail when indels exceed a certain fraction of the read length, and sometimes even miss small indels completely. Some methods rely on whole genome sequencing instead of more efficient targeted sequence capture [12–18]. About 7% of the disease-associated or functional variants in The Human Gene Mutation Database (HGMD Professional 2015.1 release) are gross indels, repeats or complex rearrangements. This is most likely an underestimate, due to difficulties in finding these changes. Nevertheless, it is important to incorporate known indels in genetic diagnostic tests.

One such challenge has been the identification of a 353 bp insertion into the *Male Germ Cell-Associated Kinase* (*MAK*) gene (MIM #154235). In 2011, distinct classes of *MAK* mutations were identified as causative mutations in retinitis pigmentosa by two different groups [19,20]. Ozgül and colleagues identified “traditional” homozygous and compound heterozygous mutations in *MAK* using whole exome sequencing and bioinformatic variant filtering (aided by gene prioritization from experimental work in mouse retinas) [19]. These variants are expected to be detected in standard NGS analysis pipelines.

However, Tucker *et al*. reported a fairly unusual class of mutation in which a 353 bp Alu repeat sequence was inserted into exon 9 of *MAK*, disrupting the gene and resulting in improper splicing and loss of the mature MAK protein [20]. It was only by serendipity that this insertion was discovered using the usual NGS bioinformatics pipelines; physical removal of repeat sequences during library preparation for ABI sequencing, combined with creation of a chimeric read led to the artifactual reporting of a “2 bp” insertion in *MAK*. After PCR amplification of the “2 bp” insertion, a much larger-than-expected fragment was observed [20]. This fragment, when Sanger sequenced, revealed a 353 bp Alu insertion. The presence of the insertion was missed completely using a GATK-based analysis pipeline on Illumina reads, since the algorithm trimmed the Alu sequence from the ends of the junction fragment reads, creating an artifactually normal *MAK* sequence [20].

Since this time, efforts have turned toward PCR-based screening of DNA from retinitis pigmentosa patients [21]. Venturini *et al*. developed a nested PCR strategy using primers to amplify exon 9 followed by an amplification using allele-specific primers, one of which contained an insertion-site junction. Using this assay in a panel of recessive retinitis pigmentosa probands, they identified the *MAK-*Alu insertion in 5/240 (2%) probands of mixed ancestry and in 9/35 (26%) probands of Jewish ancestry. Haplotype analysis confirmed that this mutation was due to a founder effect [21].

We hypothesized that the computational complexity in detection of this Alu insertion could be simplified by searching the unprocessed sequence reads for the known sequence of the mutant junction. This approach provides an attractive alternative to the complexity and resources required to implement allele-specific, nested PCR testing as part of routine genetic screening for retinitis pigmentosa. Furthermore, this computationally simple approach is appropriate for quickly screening archived NGS reads from past sequencing. These methods are of interest to clinical genetic diagnostics centers using NGS to screen patients with inherited eye diseases. Although the approach presented is very simple from a bioinformatics perspective, it solves a practical problem of missed mutation identification that is clinically relevant in current practice.

## Results

**Fig 1:**
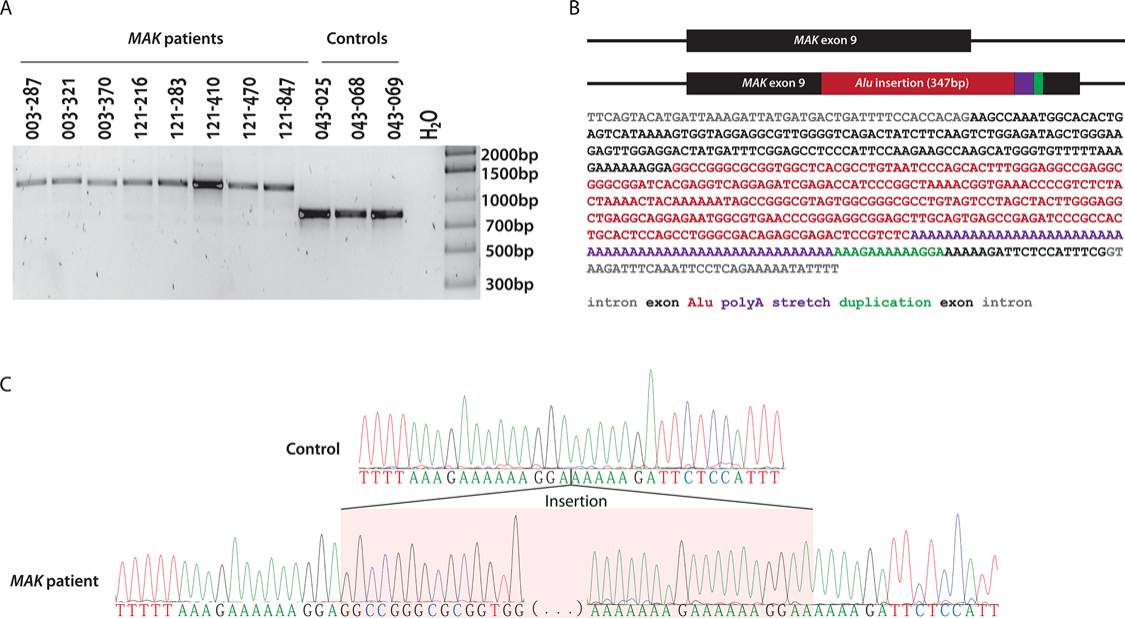
Characterization of the test sample set. A) Samples from previously reported patients with Alu insertion in *MAK* exon 9 [21] and control samples were PCR amplified to detect homozygous alleles for Alu insertion and WT alleles. B) Sequence of the inserted element (280 bp Alu, 54 bp poly-A and 13 bp duplication of exon 9 sequence). C) Sanger sequence of the exon 9 Alu insertion breakpoints.

A positive control set of DNAs known to contain the *MAK*-Alu insertion was validated. This cohort consisted of eleven samples harboring homozygous Alu insertions in *MAK* exon 9, which were previously reported [21], as well as three negative control samples (Fig 1). PCR amplification and Sanger sequencing of sample OGI412_881 revealed a 280-nucleotide Alu insertion followed by a 58 to 60-nucleotide long polyadenine stretch and a 13 bp target site duplication from *MAK* exon 9, which is a typical pattern for Alu repeat elements [22,23] (Fig 1). Bioinformatics analysis of the *MAK*-Alu insertion showed that it belongs to a relatively recently evolved AluYa8 subfamily from the class of the SINE1 non-LTR retrotransposons [24,25]. This sequence was deposited in GenBank (GenBank: KT192064).

**Fig 2:**
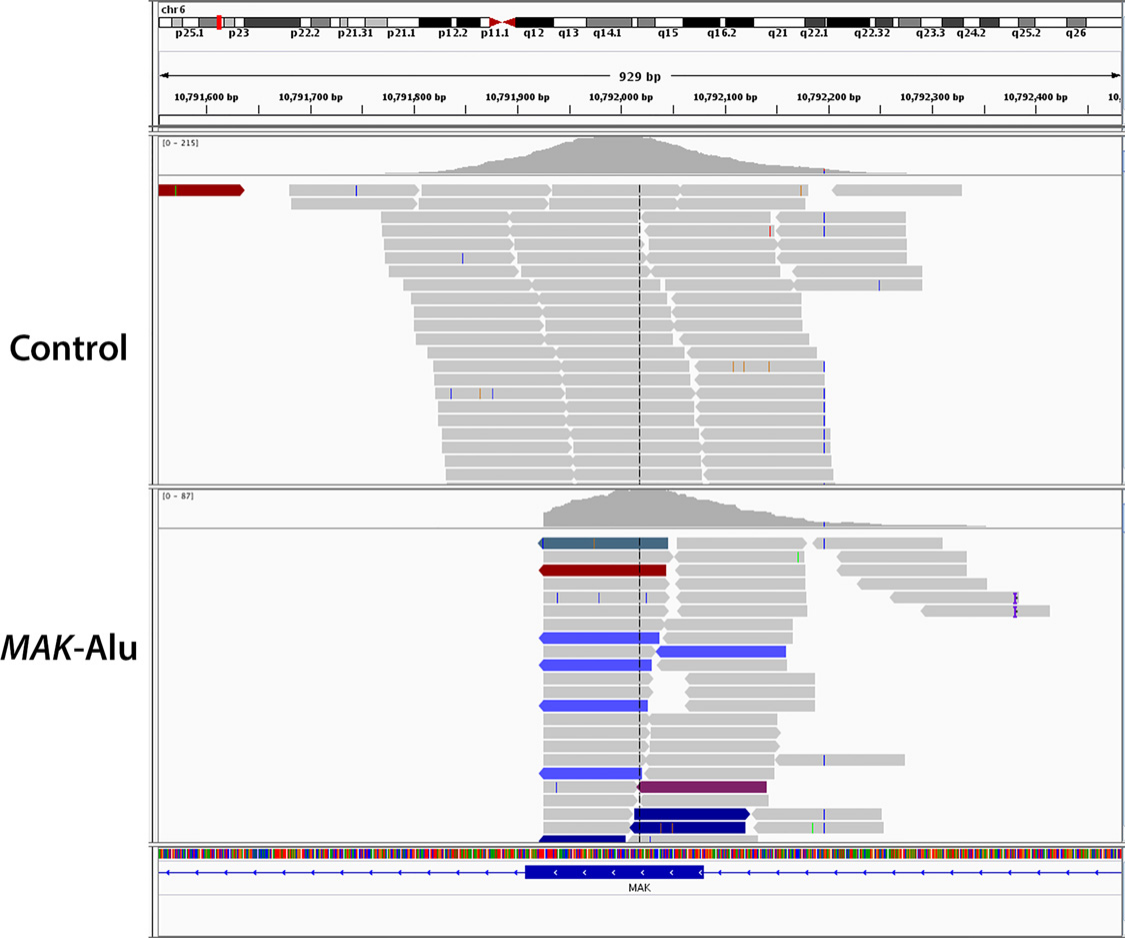
Alignment of standard BWA-based Illumina reads of a control (top) and a *MAK*-Alu homozygous (bottom) sample. *MAK*-Alu alignment produces a coverage gap in exon 9 but does not clearly identify an insertion.

In a test sample known to have a *MAK*-Alu insertion, standard BWA-based alignment of Illumina reads produces a coverage gap in *MAK* exon 9 but does not clearly identify an insertion (Fig 2). In order to improve the detection of this insertion, the Linux program *grep* was used to find Alu insertions in the unprocessed NGS reads of the test samples. All of the above test samples were NGS-sequenced using a custom targeted exon capture strategy [4] and three of the samples (003–287, 121–410 and 121–470) were also sequenced using a commercially-available whole exome sequencing protocol. Using a two-stage *grep* search algorithm (see Methods) on the FASTQ files from the targeted exon capture and the whole exome sequencing, the *MAK-*Alu insertion was detected in all positive control samples and none of the negative control samples. The reference sequence was detected in all of the negative control samples and none of the positive control samples (Table 1). These results indicate 100% sensitivity and specificity.

**Table 1:** Specificity and Sensitivity of *In Silico* Method to Detect the *MAK*-Alu insertion.

Capture / run type	Sample genotype	Sample name	Mutant junction	Mutant junction, full match	Reference junction	Reference junction, full match
**GEDi**	***MAK*-Alu**	BGL003_287	**21**	**20**	0	0
		BGL003_321	**37**	**30**	0	0
		BGL003_370	**27**	**23**	0	0
		BGL121_216	**30**	**26**	0	0
		BGL121_283	**35**	**34**	0	0
		BGL121_410	**43**	**40**	0	0
		BGL121_470	**30**	**28**	0	0
		BGL121_847	**45**	**39**	0	0
	**Control**	BGL043_067	0	0	**72**	**58**
		BGL043_068	0	0	**102**	**73**
		BGL043_069	0	0	**91**	**81**
		BGL043_072	0	0	**59**	**41**
**WES (Agilent)**	***MAK*-Alu**	BGL003_287_WES	**47**	**41**	0	0
		BGL121_410_WES	**55**	**50**	0	0
		BGL121_470_WES	**37**	**35**	7	0
	**Control**	BGL038_134	0	0	**81**	**75**
		BGL038_162	0	0	**77**	**73**
		BGL043_002	0	0	**85**	**79**
		BGL043_004	0	0	**39**	**37**
		BGL043_005	0	0	**78**	**70**
		BGL043_007	0	0	**74**	**58**
		BGL043_008	0	0	**59**	**50**
		BGL043_018	0	0	**45**	**36**
		BGL043_059	1	0	**62**	**58**
		BGL043_062	0	0	**77**	**72**
		BGL121_923	0	0	**85**	**75**
		OGI604_001255	0	0	**83**	**77**
		OGI604_001264	0	0	**82**	**77**

Testing of *in silico* method shows 100% sensitivity and specificity using custom selective exon capture data from eight known *MAK*-Alu insertion samples and four known control samples (top). Testing of *in silico* method shows 100% sensitivity and specificity using whole exome sequencing (Agilent V5+UTR) from three known *MAK*-Alu insertion samples and 13 known control samples. A “full match” requires the entire read to match an extended genomic sequence; this step removed the false positive hits in BGL121_470_WES and BGL043_059 seen in the table above (also see Methods).

An expanded version of the *grep* program was used to investigate a set of 1,847 samples, most of which are from patients with inherited retinal degenerations who were subjected to NGS-based diagnostic testing. The samples contained a mixture of targeted exon sequencing (“GEDi”) [4], whole exon sequencing and whole genome sequencing. In this cohort, five samples (from four families) were found to harbor the *MAK*-Alu insertion in exon 9 (Table 2). The true population incidence of the insertion cannot be estimated from this study, since the samples tested in this cohort had already been partially depleted of *MAK*-Alu insertion-containing samples by previous PCR-based screening [21].

**Table 2:** Identification of Homozygous and Heterozygous *MAK*-Alu Insertions in a Discovery Sample Set.

Capture / run type	Sample name	Mutant junction	Mutant junction, full match	Reference junction	Reference junction, full match	Mutant allele frequency, all matches	Mutant allele frequency, full matches	Interpretation
**Gedi**	**OGI412_881**	28	25	0	0	1	1	**homozygous mutant**
**Gedi**	**D379_148**	23	21	59	56	0.28	0.27	**heterozygous mutant**
**WES**	**C1** (relative of C2)	13	13	16	16	0.45	0.45	**heterozygous mutant**
**WES**	**C2** (relative of C1)	16	16	26	25	0.38	0.39	**heterozygous mutant**
**Gedi**	**D445_255**	13	11	15	15	0.46	0.42	**heterozygous mutant**

Analysis of NGS data from 1,847 samples efficiently identifies one homozygous *MAK-*Alu insertion and four heterozygous insertions. A “full match” requires the entire read to match an extended genomic sequence (see Methods).

**Fig 3:**
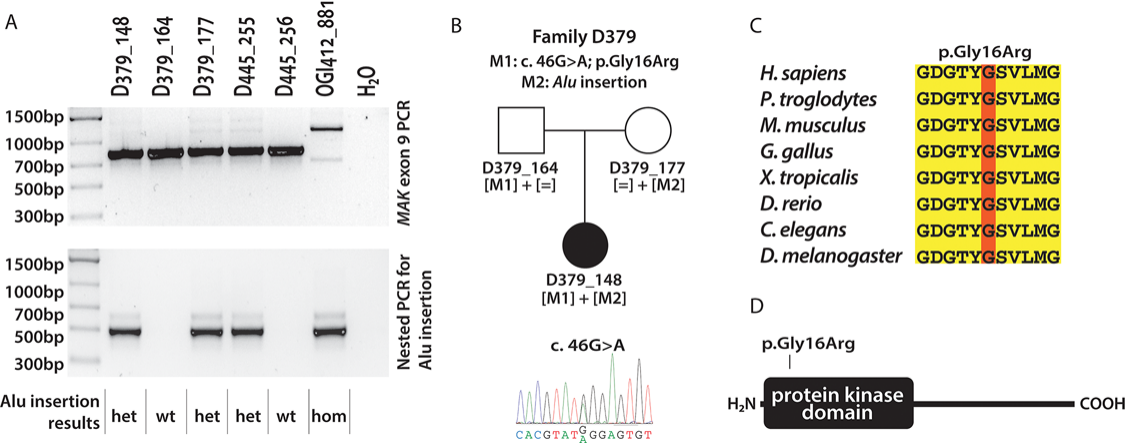
PCR validation of Alu insertion identified by *in silico* analysis in patients from the discovery cohort. A) PCR amplification using primers spanning exon 9 (top) and nested PCR using Alu-specific primer (bottom). The 1,194 bp amplicon containing the Alu insertion (arrow) is present strongly in the homozygous sample and weakly in the heterozygous samples (top); the Alu insertion-specific amplification (491 bp, bottom) confirms the presence of the Alu insertion. B) Pedigree of patient D379_148, carrying a missense mutation (p.Gly16Arg) and the Alu insertion mutation. C) Evolutionary conservation of glycine 16, mutated in the patient D379_148. D) Protein domains in MAK and location of the p.Gly16Arg change. The mutation annotations are based on the NM_001242957 transcript, where A from the ATG start codon is designated as a +1 position.

One sample was homozygous and four samples (from three families) were heterozygous for the insertion, which was confirmed in three patients by PCR (Table 2, Fig 3). The patient from family D379 (D379_148) was compound heterozygous for the *MAK*-Alu insertion and a novel missense change (c. 46G>A; p.Gly16Arg) (Fig 3A and 3B). The missense change affects a highly conserved glycine located in a protein kinase domain (Fig 3C and 3D) and is predicted to be likely pathogenic (using Polyphen-2, SIFT, Provean and MutationTaster [26–29]). The patient OGI412_881 was homozygous for the *MAK*-Alu insertion (Fig 3A); unfortunately no family members were available for co-segregation analysis. Both patients were of Ashkenazi Jewish descent, which is consistent with the *MAK*-Alu insertion being a founder mutation in this population [21]. The proband from family D445 (D445_255) carried a heterozygous *MAK*-Alu insertion (Fig 3A), however no missense changes were found in *MAK* and this patient was found to be homozygous for the c.144T>G change leading to the p.Asn48Lys substitution in *Clarin 1* (*CLRN1*), which was previously reported as a founder mutation in Usher III in the Ashkenazi Jewish population [30]. Therefore we consider D445_255 to be a heterozygous carrier of the *MAK*-Alu insertion.

The two newly-identified probands have phenotypes consistent with typical retinitis pigmentosa. The compound heterozygote patient (D379_148) had nyctalopia and constricted visual fields since her teens and was diagnosed with retinitis pigmentosa at age 25 (visual acuity 20/40 OU, visual field to a V4e test stimulus was 20 degrees diameter OU, 30 HZ ERG response of <0.2 microvolts OU). Although at age 57 her visual acuity was slightly worse due to cataracts, progression of her disease was less than expected (potential acuity meter 20/40 OD 20/60 OS, visual fields 20 degrees OU). The patient homozygous for the *MAK*-Alu insertion (OGI412_881) also had typical retinitis pigmentosa. At age 68, her visual acuity was 20/20- OU. Her visual field to a V4e test light was slightly greater than 20 degrees OU, and her 30 Hz cone ERG was 0.2 microvolts OD and 0.4 microvolts OS.

To extend this technique to other genes, a probe set was developed for the deep intronic mutation (c.2991+1655A>G) in *CEP290* [31]. Searching FASTQ file archives for this mutation, which was missed during previous versions of our full analysis pipeline, rapidly identified two samples for further attention.

## Discussion

The *MAK*-Alu *grep* program is based on knowing the sequence of the junctional insertion site, and therefore is limited to previously detected insertions (or deletions) including founder mutations in the population of interest. As an extension, additional “probe” sets can be validated for other known mutations that are not easily detectable by NGS, such as large insertions, large deletions, or other large rearrangements. Of note, our in-house NGS pipeline has an indel detection limit of approximately 30–50 nucleotides depending on the sequence length and quality.

We have optimized the probe set and reference sequences for the *MAK*-Alu insertion, which is described in the Methods section and available as part of the downloadable program [32]. Other researchers who find additional non-mapping insertions or deletions of interest are welcome to contact the authors or submit them to the above website.

For mutations that *are* easily detectable by standard NGS pipelines, this method may occasionally by useful as well. For example, the fact that this method works on.FASTQ or compressed.FASTQ files makes it appropriate for quickly searching archived sequence reads without having to use the computational time and storage to extract archived sequence data, recreate alignments, and recreate variant call files, as shown for the *CEP290* deep intronic mutation.

Using this method requires *a priori* knowledge of the sequence at one of the insertion’s junctions, and that newly formed junction must not already exist in the genome. There has been significant work on more general methods of detection of chromosomal breakpoints and insertions [12]. The *MAK*-Alu insertion is a particularly difficult subset of “breakpoint” or “chimeric read” to clearly identify, since half of the read is non-mapping due to being a repeat sequence. *De novo* detection of such sequences is an area for future study. Until those methods are perfected, the simplicity and computational efficiency of searching for the junction sequence is advantageous and effective in practice.

## Conclusions

The *MAK*-Alu insertion was discovered by happenstance [20], as it normally does not show up in typical NGS analysis pipelines, including our own. The need to do a separate PCR to detect this mutation is relatively time-consuming and costly. For this reason it is advantageous to detect the Alu insertion using the *MAK*-Alu *grep* program on the NGS data. Using a discovery set of 1,847 samples, the efficient *in silico* algorithm presented here identified *MAK*-Alu insertions in five samples and we showed that this technique has high specificity and sensitivity. This approach, while quite simple from a bioinformatics perspective, can be of immediate practical use to clinical diagnostic laboratories that use NGS, until such time as improved NGS processing pipelines no longer miss such clinically-important insertions. The downloadable software is pre-configured to detect the *MAK*-Alu insertion that is applicable to inherited eye diseases, but is modifiable to detect other known genomic insertions, deletions, and rearrangements from this or other disease areas.

## Methods

### Patient cohort

The study protocol adhered to the tenets of the Declaration of Helsinki and was approved by the Institutional Review Boards of Massachusetts Eye and Ear Infirmary and Harvard Medical School. The patients harboring the *MAK*-Alu insertion in the test sample set (Fig 1) were previously reported by Venturini and colleagues [21]. To our knowledge all probands were unrelated. The patients with clinical information included in the study were recruited and clinically examined at the Massachusetts Eye and Ear Infirmary. After patients signed consent forms, blood samples were collected from patients for DNA extraction.

### Identification of *MAK*-Alu insertion in NGS reads

The Linux program *grep* was used to search FASTQ files for the 5’ junction between the reference sequence of exon 9 and the beginning of the Alu insertion. This is the same sequence used as an allele-specific primer by Venturini et al. [21]. A full software implementation is available online [32]. For the purpose of explanation, at its simplest, the approach can be implemented as follows:

grep–c GAAAAAAGGAGGCCGGGCGCGGT sequence.fastq

This returns the number of reads containing the mutant junction in that sequence file. (An example of the matching raw reads are shown in S1 Fig) Modifications to search compressed files, detect the reverse complement in unoriented reads, and to detect the reference/wildtype sequence are:

zgrep–c ACCGCGCCCGGCCTCCTTTTTTC\|GAAAAAAGGAGGCCGGGCGCGGT sequence.fastq.gz > mutantcount

zgrep–c CGAAATGGAGAATCTTTTTTCCT\|AGGAAAAAAGATTCTCCATTTCG sequence.fastq.gz > wildtypecount

In a *MAK*-Alu-containing sample, the program returns a positive value depending on the coverage depth in that area (typically 21–55 reads but as low as 13—see Tables 1 and 2). Most files without the insertion return a count of “0” though rarely a false-positive read count of 1 or 2 was detected in a minority of wildtype samples (36/1,847 = 1.9%). In one negative control sample which was run on the same flowcell as a positive sample, seven false positive reads were obtained. A cutoff could be established to exclude such samples with small numbers of hits (e.g. in the grepsearch software, a count of 1 or 2 mutant reads are flagged as probable false positives). Alternatively, the raw read count could be normalized to the total number of reads in the sample; this resulted in a metric that was actually worse at distinguishing true positives from false positives. Instead, a second computational step was implemented to eliminate such false-positive reads automatically. A second level of *grep* screening was performed where each read matching the probe sequence was further compared to a “reference” sequence spanning the insertion site of ~300 bp of reference genomic DNA. For the mutant “reference” sequence, we included the Alu insertion as described above. The number of reads that match both the probe sequence and the full reference sequence are referred to as a “full match” in the tables. Because using the extended reference sequence eliminated all false positive hits, there is no longer a need to flag/exclude counts of only 1 or 2 mutant reads. The requirement to exactly match the extended reference sequence, as currently implemented, has the disadvantage that, theoretically, a second-site SNP near the junction could prevent matching the full “reference” sequence; this false-negative result was not observed in the current data sets and is probably rare in this haplotype.

Scripts to run these tests on batches of FASTQ files are provided online [32].

### PCR validation of *MAK-*Alu insertion

The validation for *MAK*-Alu insertion was performed by PCR using the previously reported primers: 5′-TACCGCCCATTTTTGTTCAT-3′ (intron 8, forward) and 5′-ACTGAGAACTGTTACTGTGAG-3′ (intron 9, reverse) [21]. The PCR reaction was performed using a 5x PCR polymerase master mix (5x HOT FIREPol® Blend Master Mix with 7.5 MgCl_2_, Solis Biodyne, Estonia), using 20ng of genomic DNA and 0.3μM of each primer in a 20μl reaction. The amplification conditions were the following: 95°C for 10 minutes; 35 cycles of 95°C for 30 seconds, 60°C for 30 seconds and 72°C for 1 minute; final extension at 72°C for 5 minutes. Since the PCR reaction preferentially amplifies the shorter WT allele in samples with a heterozygous Alu insertion, a nested PCR was performed using the following primers: 5’-GAAAAAAGGAGGCCGGGCGCGGT-3’(Alu nested [21]) and 5’-CCTGGCCTGTTAAGCAAACT-3’ (reverse nested). The same PCR reaction conditions were used, except for shortening of the extension time to 30 seconds and reducing the cycle number to 25.

Sanger sequencing was performed after PCR cleanup (ExoSap-IT, Affymetrix, Santa Clara, CA, USA) and sequenced (BigDye Terminator v3.1, ABI 3730xl, Life Technologies, Grand Island, NY, USA) using the intron 8 and intron 9 primers described above.

### Custom selective exon capture, whole exome sequencing (WES), and next generation sequencing (NGS)

For custom selective exon capture, paired-end/multiplexable SureSelect targeted enrichment capture libraries (Agilent Technologies, Inc.; Santa Clara, CA) were generated on a BRAVO automation workstation (Agilent Technologies, Inc.) according to their standard automation protocol (Pub. No. G7550-90000, Version D.1, April 2012). Targeted enrichment included all currently known monogenic inherited retinal degeneration genes [3,4]. Targeted enrichment sample analysis was performed on a MiSeq NGS platform (Illumina, Inc.; San Diego, CA). A 12-patient sample multiplex was clustered to an average cluster density of ~850 K clusters per mm^2^ and 2 x 121 bp paired-end sequenced using Illumina’s 300-cycle MiSeq Reagent Kit V2. The average depth-of-coverage (DoC) per-sample was ~100x.

WES targeted enrichment capture libraries were generated on a BRAVO automated workstation using the SureSelect Human V5+UTR All Exon targeted enrichment kit (Agilent Technologies, Inc.) according to their standard automation protocol. NGS analysis was performed using a HiSeq 2500 NGS instrument (Illumina, Inc.) in the High Throughput mode. An 8 picoMolar (pM), 4-sample multiplex sample (i.e. 2 pM per capture library) was clustered in duplicate flow cell lanes at ~700,000 clusters per mm^2^, followed by 101|7|101 bp paired-end indexed analysis. The average DoC for the sixteen WES samples was 67x; additionally, the average percent on-target coverage for these WES samples at 1x, 10x, and 20x DoC was 99.9%, 92.2% and 80.9%, respectively.

## Supporting Information

S1 Fig Matching mutant raw reads example. Using the command “zgrep GAAAAAAGGAGGCCGGGCGCGGT D00379_000148_GCCAAT_L001_R2_001.fastq.gz”, 23 reads were obtained. The reads were aligned manually for display purposes and the sequence matching the probe was underlined. A space was added before the canonical 5’ end of the Alu insertion (GGCCGGG…). The read length of 121 bp was too short to span the entire Alu insertion (even if each read was computationally merged with its mate pair, not shown).
(DOCX)
