# Supplementary material for: Efficient In Silico Identification of a Common Insertion in the MAK Gene which Causes Retinitis Pigmentosa
Source: PLoS One. 2015 Nov 11;10(11):e0142614. doi: 10.1371/journal.pone.0142614 (PMC4641726; doi:10.1371/journal.pone.0142614)
Supplement: S1 Fig — Using the command “zgrep GAAAAAAGGAGGCCGGGCGCGGT D00379_000148_GCCAAT_L001_R2_001.fastq.gz”, 23 reads were obtained. The reads were aligned manually for display purposes and the sequence matching the probe was underlined. A space was added before the canonical 5’ end of the Alu insertion (GGCCGGG…). The read length of 121 bp was too short to span the entire Alu insertion (even if each read was computationally merged with its mate pair, not shown). (DOCX) [file pone.0142614.s001.docx]

**AAGAAAAAAGGA GGCCGGGCGCGGTGGCTCACGCCTGTAATCCCAGCACTTTGGGAGGCCGAGGCGGGCGGATCACGAGGTCAGGAGATCGAGACCATCCCGGCTAAAACGGTGAAACCCC**

**ATCTTCAAGTCTGGAGATAGCTGGGAAGAGTTGGAGGACTATGATTTCGGAGCCTCCCATTCCAAGAAGCCAAGCATGGGTGTTTTTAAAGAAAAAAGGA GGCCGGGCGCGGTGGCTCACG**

**AAGAAGCCAAGCATGGGTGTTTTTAAAGAAAAAAGGA GGCCGGGCGCGGTGGCTCACGCCTGTAATCCCAGCACTTTGGGAGGCCGAGGCGGGCGGATCACGAGGTCAGGAGATCGAGACC**

**GAAGCCAAGCATGGGTGTTTTTAAAGAAAAAAGGA GGCCGGGCGCGGTGGCTCACGCCTGTAATCCCAGCACTTTGGGAGGCCGAGGCGGGCGGATCACGAGGTCAGGAGATCGAGACCAT**

**CAAGTCTGGAGATAGCTGGGAAGAGTTGGAGGACTATGATTTCGGAGCCTCCCATTCCAAGAAGCCAAGCATGGGTGTTTTTAAAGAAAAAAGGA GGCCGGGCGCGGTGGCTCACGCCTGT**

**GAAGCCAAGCATGGGTGTTTTTAAAGAAAAAAGGA GGCCGGGCGCGGTGGCTCACGCCTGTAATCCCAGCACTTTGGGAGGCCGAGGCGGGCGGATCACGAGGTCAGGAGATCGAGACCAT**

**GAGTTGGAGGACTATGATTTCGGAGCCTCCCATTCCAAGAAGCCAAGCATGGGTGTTTTTAAAGAAAAAAGGA GGCCGGGCGCGGTGGCTCACGCCTGTAATCCCAGCACTTTGGGAGGCC**

**TATCTTCAAGTCTGGAGATAGCTGGGAAGAGTTGGAGGACTATGATTTCGGAGCCTCCCATTCCAAGAAGCCAAGCATGGGTGTTTTTAAAGAAAAAAGGA GGCCGGGCGCGGTGGCTCAC**

**CAAGTCTGGAGATAGCTGGGAAGAGTTGGAGGACTATGATTTCGTAGCCTCCCATTCCAAGAAGCCAAGCATGGGGGTTTTTAAAGAAAAAAGGA GGCCGGGCGCGGTGGCTCACGCCTGT**

**ATCTTCAAGTCTGGAGATAGCTGGGAAGAGTTGGAGGACTATGATTTCGGAGCCTCCCATTCCAAGAAGCCAAGCATGGGTGTTTTTAAAGAAAAAAGGA GGCCGGGCGCGGTGGCTCACG**

**TATCTTCAAGTCTGGAGATAGCTGGGAAGAGTTGGAGGACTATGATTTCGGAGCCTCCCATTCCAAGAAGCCAAGCATGGGTGTTTTTAAAGAAAAAAGGA GGCCGGGCGCGGTGGCTCAC**

**GCCTCCCATTCCAAGAAGCCAAGCATGGGTGTTTTTAAAGAAAAAAGGA GGCCGGGCGCGGTGGCTCACGCCTGTAATCCCAGCACTTTGGGAGGCCGAGGCGGGCGGATCACGAGGTCAG**

**CAAGTCTGGAGATAGCTGGGAAGAGTTGGAGGACTATGATTTCGGAGCCTCCCATTCCAAGAAGCCAAGCATGGGTGTTTTTAAAGAAAAAAGGA GGCCGGGCGCGGTGGCTCACGCCTGT**

**CATTCCAAGAAGCCAAGCATGGGTGTTTTTAAAGAAAAAAGGA GGCCGGGCGCGGTGGCTCACGCCTGTAATCCCAGCACTTTGGGAGGCCGAGGCGGGCGGATCACGAGGTCAGGAGATC**

**ATTCCAAGAAGCCAAGCATGGGTGTTTTTAAAGAAAAAAGGA GGCCGGGCGCGGTGGCTCACGCCTGTAATCCCAGCACTTTGGGAGGCCGAGGCGGGCGGATCACGAGGTCAGGAGATCG**

**AAGCATGGGTGTTTTTAAAGAAAAAAGGA GGCCGGGCGCGGTGGCTCACGCCTGTAATCCCAGCACTTTGGGAGGCCGAGGCGGGCGGATCACGAGGTCAGGAGATCGAGACCATCCCGGC**

**AGTTGGAGGACTATGATTTCGGAGCCTCCCATTCCAAGAAGCCAAGCATGGGTGTTTTTAAAGAAAAAAGGA GGCCGGGCGCGGTGGCTCACGCCTGTAATCCCAGCACTTTGGGAGGCCG**

**CTTCAAGTCTGGAGATAGCTGGGAAGAGTTGGAGGACTATGATTTCGGAGCCTCCCATTCCAAGAAGCCAAGCATGGGTGTTTTTAAAGAAAAAAGGA GGCCGGGCGCGGTGGCTCACGCC**

**GGGTGTTTTTAAAGAAAAAAGGA GGCCGGGCGCGGTGGCTCACGCCTGTAATCCCAGCACTTTGGGAGGCCGAGGCGGGCGGATCGCGAGGTCAGGAGATCGAGACCATCCCGGCTAAAAC**

**TTTTTAAAGAAAAAAGGA GGCCGGGCGCGGTGGCTCACGCCTGTAATCCCAGCACTTTGGGAGGCCGAGGCGGGCGGATCACGAGGTCAGGAGATCGAGACCATCCCGGCTAAAACGGTGA**

**ATCTTCAAGTCTGGAGATAGCTGGGAAGAGTTGGAGGACTATGATTTCGGAGCCTCCCATTCCAAGAAGCCAAGCATGGGTGTTTTTAAAGAAAAAAGGA GGCCGGGCGCGGTGGCTCACG**

**AGAGTTGGAGGACTATGATTTCGGAGCCTCCCATTCCAAGAAGCCAAGCATGGGTGTTTTTAAAGAAAAAAGGA GGCCGGGCGCGGTGGCTCACGCCTGTAATCCCAGCACTTTGGGAGGC**

**AGAGTTGGAGGACTATGATTTCGGAGCCTCCCATTCCAAGAAGCCAAGCATGGGTGTTTTTAAAGAAAAAAGGA GGCCGGGCGCGGTGGCTCACGCCTGTAATCCCAGCACTTTGGGAGGC**

**S1 Fig.** **Matching mutant raw reads example.** Using the command “zgrep GAAAAAAGGAGGCCGGGCGCGGT D00379_000148_GCCAAT_L001_R2_001.fastq.gz”, 23 reads were obtained. The reads were aligned manually for display purposes and the sequence matching the probe was underlined. A space was added before the canonical 5’ end of the Alu insertion (GGCCGGG…). The read length of 121 bp was too short to span the entire Alu insertion (even if each read was computationally merged with its mate pair, not shown).
